# The Induction of MicroRNA Targeting IRS-1 Is Involved in the Development of Insulin Resistance under Conditions of Mitochondrial Dysfunction in Hepatocytes

**DOI:** 10.1371/journal.pone.0017343

**Published:** 2011-03-25

**Authors:** Hyun Su Ryu, Seung-Yoon Park, Duan Ma, Jin Zhang, Wan Lee

**Affiliations:** 1 Department of Biochemistry, Dongguk University College of Medicine, Kyungju, Korea; 2 Key Lab of Molecular Medicine, Shanghai Medical College, Institutes of Biomedical Sciences, Fudan University, Shanghai, China; University of Las Palmas de Gran Canaria, Spain

## Abstract

**Background:**

Mitochondrial dysfunction induces insulin resistance in myocytes via a reduction of insulin receptor substrate-1 (IRS-1) expression. However, the effect of mitochondrial dysfunction on insulin sensitivity is not understood well in hepatocytes. Although research has implicated the translational repression of target genes by endogenous non-coding microRNAs (miRNA) in the pathogenesis of various diseases, the identity and role of the miRNAs that are involved in the development of insulin resistance also remain largely unknown.

**Methodology:**

To determine whether mitochondrial dysfunction induced by genetic or metabolic inhibition causes insulin resistance in hepatocytes, we analyzed the expression and insulin-stimulated phosphorylation of insulin signaling intermediates in SK-Hep1 hepatocytes. We used *q*RT-PCR to measure cellular levels of selected miRNAs that are thought to target *IRS-1* 3′ untranslated regions (3′UTR). Using overexpression of miR-126, we determined whether *IRS-1*-targeting miRNA causes insulin resistance in hepatocytes.

**Principal Findings:**

Mitochondrial dysfunction resulting from genetic (mitochondrial DNA depletion) or metabolic inhibition (Rotenone or Antimycin A) induced insulin resistance in hepatocytes via a reduction in the expression of IRS-1 protein. In addition, we observed a significant up-regulation of several miRNAs presumed to target *IRS-1* 3′UTR in hepatocytes with mitochondrial dysfunction. Using reporter gene assay we confirmed that miR-126 directly targeted to *IRS-1* 3′UTR. Furthermore, the overexpression of miR-126 in hepatocytes caused a substantial reduction in IRS-1 protein expression, and a consequent impairment in insulin signaling.

**Conclusions/Significance:**

We demonstrated that miR-126 was actively involved in the development of insulin resistance induced by mitochondrial dysfunction. These data provide novel insights into the molecular basis of insulin resistance, and implicate miRNA in the development of metabolic disease.

## Introduction

Insulin resistance is defined as the decreased responsiveness of target tissues to ordinary levels of insulin and plays a central role in the development of metabolic disorders such as type 2 diabetes, hypertension, and dyslipidemia [1], [2]. A number of studies have provided support for the hypothesis that genetic or functional impairments in mitochondria are involved in the development of insulin resistance [3], [4]. Cellular oxidative capacity, which mostly depends on mitochondrial function, is directly correlated with insulin sensitivity in skeletal muscles [5], [6], [7], and reduced mitochondria activity has been observed in patients with obesity and type 2 diabetes [8], [9], [10]. Although, several investigations demonstrated that skeletal muscle oxidative capacity and mitochondrial function are not a primary factor for insulin sensitivity in obese subjects [11], [12], [13], [14], emerging evidence support that mitochondrial dysfunction may play an important role in the pathogenesis of insulin resistance and type 2 diabetes [15], [16], [17]. Recently, it has been suggested that mitochondrial dysfunction induced by inhibitors of mitochondrial function or depletion of mitochondrial DNA (mtDNA) causes insulin resistance in myocytes through a reduction in the expression of insulin receptor substrate (IRS)-1, a protein with a pivotal role in the insulin signaling cascade [18], [19].

IRS-1 is a key molecule in insulin signaling, and is involved in signal transduction between the insulin receptor and phosphoinositide 3-kinase (PI3K)[20]. Several lines of evidence suggest that a reduction in IRS-1 protein plays an important role in the development of insulin resistance and type 2 diabetes. IRS-1 is decreased in skeletal muscle and liver of animal models established for insulin resistance and type 2 diabetes, such as *ob/ob* mice [21], [22] and Zucker fatty rats [23]. Studies have also reported decreased IRS-1 expression in the skeletal muscle of patients with diabetes, and concluded that this could represent a marker for the risk of insulin resistance [24], [25], [26], [27]. However, the molecular mechanism underlying the reduction of IRS-1 expression in muscle and liver under conditions of mitochondrial dysfunction remains largely unknown.

MicroRNAs (miRNAs) are endogenous small non-coding RNAs that act as posttranscriptional regulators [28]. Mature miRNAs hybridize to partially complementary binding sites that are typically localized in the 3′ untranslated regions (3′UTR) of target mRNAs [29]. This property allows a single miRNA sequence to have multiple target sites on various mRNAs. Upon binding, the miRNA initiates a pathway that either degrades the transcripts or suppresses its translation [29]. Although the target genes and biological functions of individual miRNAs remain largely unknown, it has been suggested that miRNAs have diverse functions in both normal and pathological states [28]. Recent research has established that unregulated miRNAs expression is implicated in defective insulin secretion, diabetic kidney and heart disease [30]. However, no previous study has investigated the involvement of miRNAs in metabolic disorders, in particular the development of insulin resistance.

In the present study, we demonstrated that mitochondrial dysfunction resulting from genetic or metabolic inhibition provoked insulin resistance via a decrease in the expression of IRS-1. Furthermore, we found that mitochondrial dysfunction induced the expression of several miRNAs thought to target the *IRS-1* 3′UTR and that miR-126 was actively involved in the development of insulin resistance. Our findings in hepatocytes reveal a novel mechanism for the development of insulin resistance by providing the first direct evidence that miR-126 mediates the repression of IRS-1 expression in mitochondrial dysfunction.

## Results

**Figure 1:**
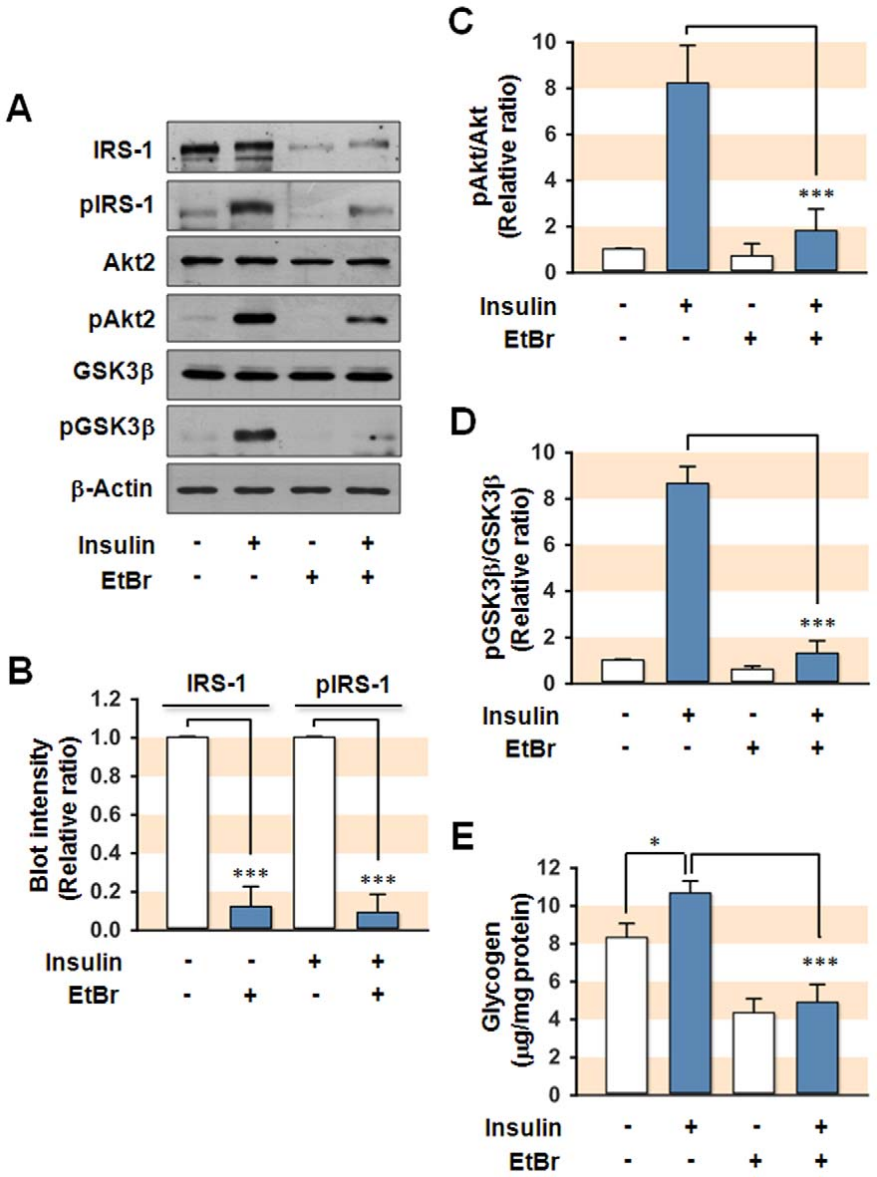
Effect of mtDNA depletion induced by EtBr on insulin signaling. SK-Hep1 hepatocytes depleted of mtDNA was prepared by EtBr treatment for 2 weeks. Cells were preincubated in the absence or presence of insulin (100 nM for 20 min) and total cell lysates (15 µg protein) were subjected to 8% SDS-PAGE. (**A**) The expression (IRS-1, Akt2, and GSK3β) and phosphorylation (pIRS-1, pAkt2, and pGSK3β) of insulin signaling intermediates were analyzed by immunoblot. (**B**) The immunoblot intensities for IRS-1/β-Actin (IRS-1) and insulin-stimulated pIRS-1/β-Actin (pIRS-1) were quantified by densitometry and expressed in relative ratio where the intensity of normal control was set to one. (**C–D**) The immunoblot intensities for pAkt2/Akt2 and pGSK3β/GSK3β were quantified by densitometry and expressed in relative ratio. The intensity of normal control was set to one. (**E**) Cellular glycogen contents were analyzed by spectrophotometry (570 nm) with OxiRed probe. Values are expressed as means ± SEM from at least five independent experiments.*, P<0.05; ***, P<0.001.

Hepatocytes depleted of mtDNA were prepared by exposing SK-Hep1 hepatocytes to a low dose of ethidium bromide (EtBr, 0.2 µg/ml) for two weeks, as described previously for myocytes [18]. As shown in Figure S1, treatment with EtBr depleted mtDNA without altering nuclear DNA, and provoked mitochondrial dysfunction. To determine whether mitochondrial dysfunction induced by mtDNA depletion causes insulin resistance in hepatocytes, we analyzed the expression and insulin-stimulated phosphorylation of insulin signaling intermediates, such as IRS-1, Akt2, and glycogen synthase kinase-3β (GSK3β), in the mtDNA-depleted SK-Hep1 hepatocytes. We observed about 90% reduction in the expression of IRS-1 in the cells depleted of mtDNA by EtBr treatment (Figure 1*A*-*B*). As expected, mtDNA depletion significantly reduced the phosphorylation of IRS-1 and its substrate, Akt2 (Figure 1*B*–*C*). We also found a significant reduction in the insulin-stimulated phosphorylation of GSK3β, a substrate of Akt2, in the mtDNA-depleted hepatocytes compared to control hepatocytes (Figure 1*D*). In addition, cellular glycogen contents in the presence or absence of insulin were analyzed in the control and mtDNA-depleted cells (Figure 1*E*). As we expected, insulin significantly increased glycogen contents in control cells. However, the depletion of mtDNA by EtBr significantly reduced basal glycogen content as compared to control. Moreover, insulin-stimulated glycogen synthesis was almost completely disappeared in mtDNA-depleted hepatocytes (Figure 1*E*). These findings clearly indicate that mitochondrial dysfunction resulting from mtDNA depletion induces the development of insulin resistance in hepatocytes through a reduction in the expression of IRS-1.

**Figure 2:**
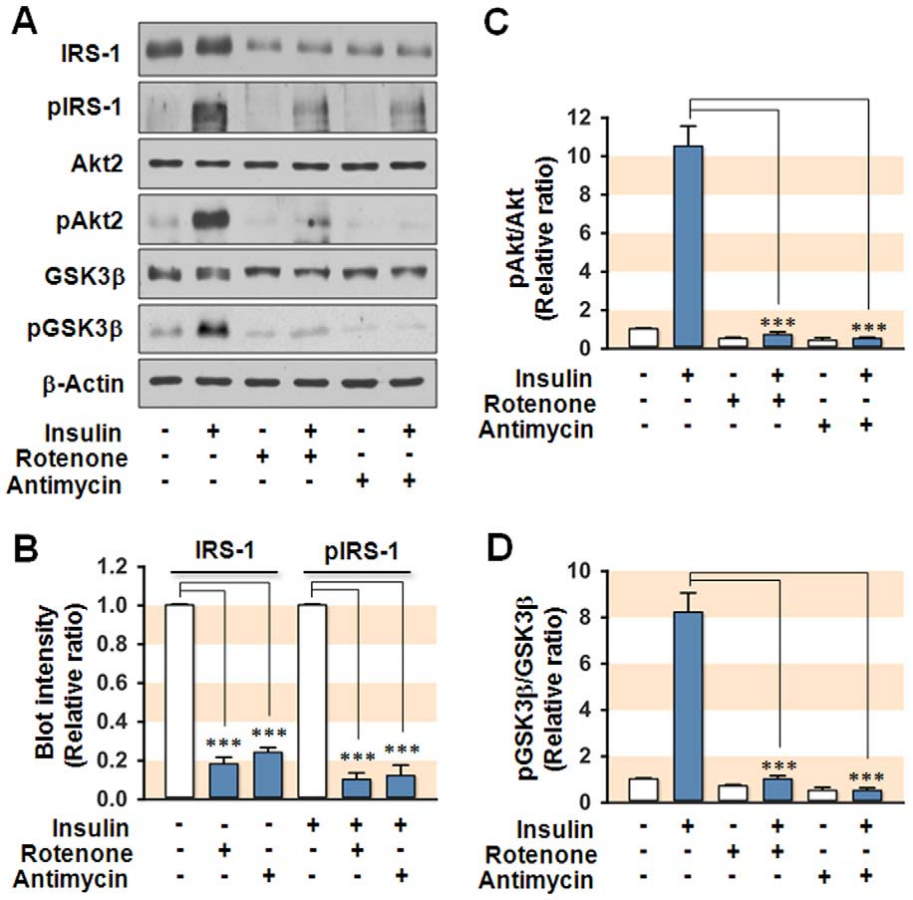
Effect of mitochondrial metabolic inhibitors on insulin signaling. For mitochondrial metabolic inhibition, hepatocytes were treated with Rotenone (0.1 µM) or Antimycin A (20 µM) for 18 h. Cells were incubated in the absence or presence of insulin (100 nM for 20 min) and total cell lysates (15 µg protein) were subjected to 8% SDS-PAGE. (**A**) The expression (IRS-1, Akt2, and GSK3β) and phosphorylation (pIRS-1, pAkt2, and p GSK3β) of insulin signaling intermediates were analyzed by immunoblot. (**B**) The immunoblot intensities for IRS-1/β-Actin (IRS-1) and insulin-stimulated pIRS-1/β-Actin (pIRS-1) were quantified by densitometry and expressed in relative ratio where the intensity of normal control was set to one. (**C–D**) The immunoblot intensities for pAkt2/Akt2 and pGSK3β/GSK3β were quantified by densitometry and expressed in relative ratio. The intensity of normal control was set to one. Values are expressed as means ± SEM from at least five independent experiments.***, P<0.001.

We then investigated whether mitochondrial dysfunction resulting from metabolic inhibitors, such as Rotenone (an inhibitor of electron transfer from complex I to ubiquinone) and Antimycin A (an inhibitor of complex III), causes impairment of insulin signaling in SK-Hep1 hepatocytes. We observed an almost complete loss of functional mitochondria in the hepatocytes treated with Rotenone or Antimycin A (Figure S2), whereas the level of mtDNA and its transcripts were not affected by inhibitors (data not shown). Interestingly, treatment with Rotenone or Antimycin A caused a significant reduction in the expression of IRS-1, whereas the expression of both Akt2 and GSK3β remained unaffected as compared to control (Figure 2*A*–*B*). Both inhibitors caused a significant reduction in the insulin-stimulated phosphorylation of IRS-1, Akt2, and GSK3β (Figure 2*B*–*D*). These findings suggest that mitochondrial dysfunction secondary to metabolic inhibition induces the development of insulin resistance in hepatocytes by decreasing the expression of IRS-1.

**Figure 3:**
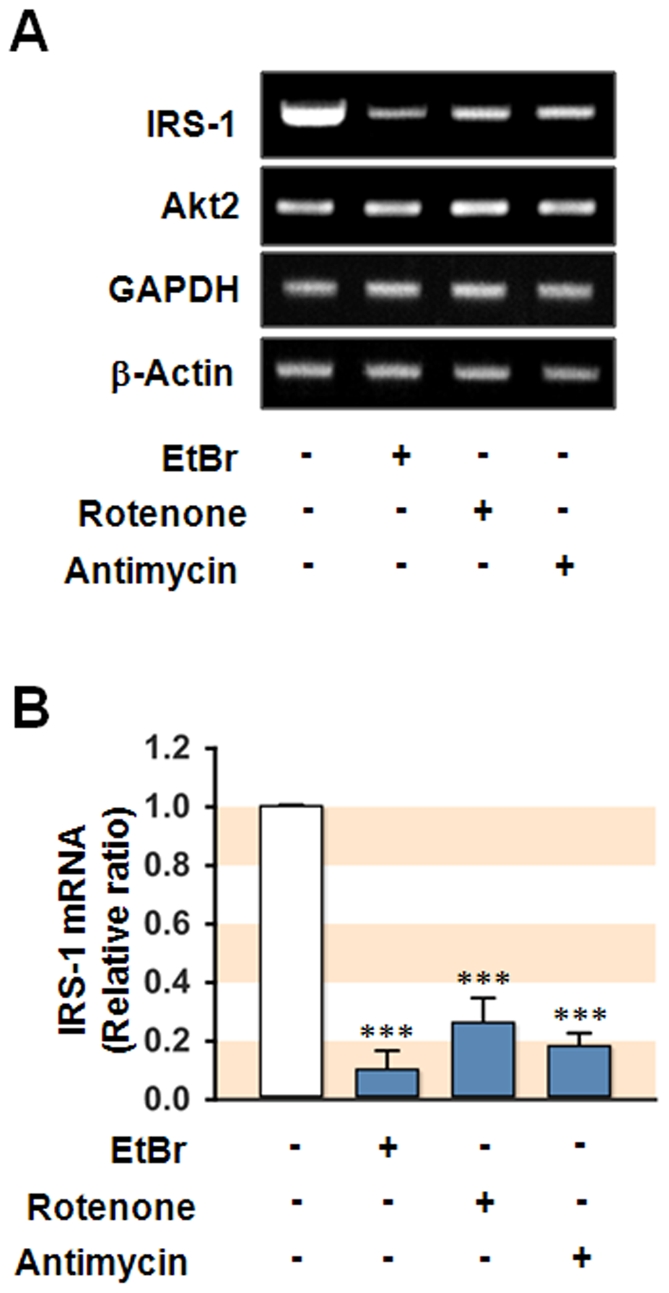
Transcriptional inhibition of IRS-1 in hepatocytes with dysfunctional mitochondria. SK-Hep1 hepatocytes were treated with vehicle, EtBr (0.2 µg/ml, 2 week), Rotenone (0.1 µM, 18 h), or Antimycin A (20 µM, 18 h), and the expression levels of IRS-1, Akt2, GAPDH, and β-Actin were measured by (**A**) RT-PCR and (**B**) *q*RT-PCR. The fold changes are expressed in relative ratio where the intensity of β-Actin was set to one. All results represent mean ± SEM from three independent experiments. ***, P<0.001 vs Control.

**Figure 4:**
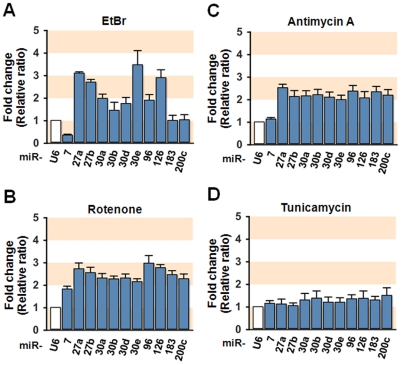
Expression of miRNAs presumably targeting *IRS-1* 3′UTR. SK-Hep1 hepatocytes were treated with (**A**) EtBr (0.2 µg/ml, 2 week), (**B**) Rotenone (0.1 µM, 18 h), (**C**) Antimycin A (20 µM, 18 h), or (**D**) Tunicamycin (2 µg/ml, 18 h), and the expression levels of miRNAs that predicted to target *IRS-1* 3′UTR were measured by *q*RT-PCR. The fold changes are expressed in relative ratio where U6 intensity set to one.

Although mitochondrial dysfunction provoked a substantial decline of IRS-1 mRNA as shown in Figure 3*A–B*, posttranscriptional repression of IRS-1 should not be excluded. Therefore, to determine whether miRNA induces repression of IRS-1 expression posttranscriptionally, we selected several miRNAs targeting *IRS-1* and measured cellular levels of these miRNAs in hepatocytes with dysfunctional mitochondria. Although mitochondrial dysfunction causes numerous changes in the expression of miRNAs in SK-Hep1 cells, we initially selected miRNAs presumably targeting a segment of *IRS-1* 3′UTR (within 500 nucleotides from the stop codon) by TargetScan, MiBase, and PicTar analysis. These analyses revealed that about 70 miRNAs presumably targeting this region of *IRS-1* 3′UTR. Subsequently, as shown in Table S1, we selected 11 miRNAs for the next experiments according to its targeting score to *IRS-1* 3′UTR, as well as quality and quantity of the results from *q*RT-PCR analysis. Although it has been reported that miR-145 targets IRS-1 in colon cancer cells [31], we excluded miR-145 in this study because of its low expression level. Interestingly, mtDNA depletion significantly (more than 2-fold, P<0.05) increased the levels of several of these miRNAs including miR-27a, miR-27b, miR-30e, and miR-126, whereas the levels of other selected miRNAs remained unaffected (Figure 4*A*). In experiments in which mitochondrial dysfunction was induced by Rotenone (Figure 4*B*) or Antimycin A (Figure 4*C*), a significant increase in the levels of all of the selected miRNAs was observed, with the exception of miR-7. Tunicamycin is well known to induce endoplasmic reticulumn (ER) stress in various cells including SK-Hep1 hepatocytes. Although treatment with tunicamycin (2 µg/ml) induced ER stress assessed by phosphorylation of eIF2α (Figure S3), tunicamycin resulted in no significant change in the levels of any of the 11 selected miRNAs (Figure 4*D*) or the expression of IRS-1 (Figure S3). In addition, the levels of miRNAs such as miR-137, miR-663, miR-1231, miR-1246, and miR-1287, which are not targeting *IRS-1* 3′UTR, were not affected by mitochondrial dysfunction (data not shown). This result indicates that mitochondrial dysfunction increases the level of several miRNAs that are predicted to target *IRS-1* 3′UTR, thus potentially implicating miRNAs in the development of insulin resistance.

**Figure 5:**
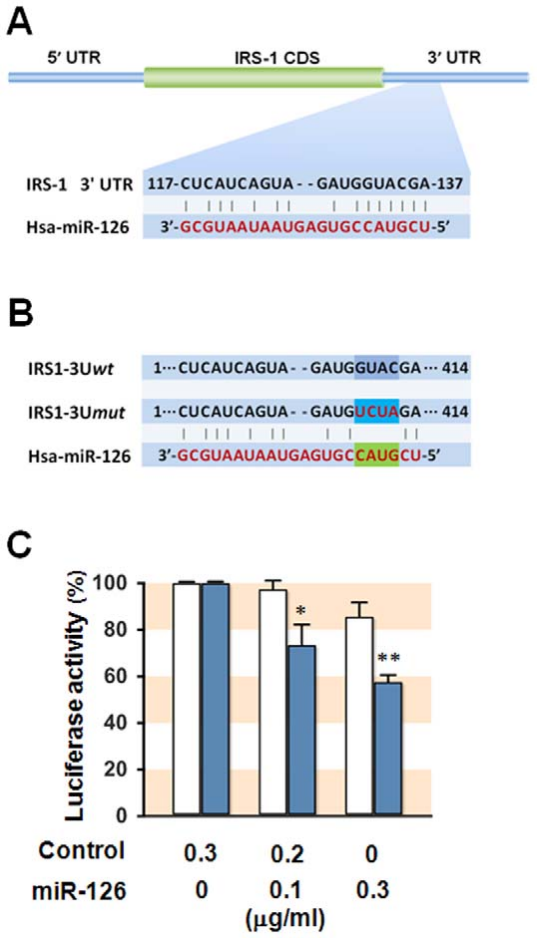
Targeting site of miR-126 in the 3′UTR of *IRS-1* and measurement of its binding by reporter gene assay. (**A**) Seed sequence of miR-126 is predicted to target *IRS-1* 3′UTR. (**B**) For reporter gene assay, 414 nt-long 3′UTR of the *IRS-1* gene was inserted downstream of a luciferase open reading frame (IRS1-3U*wt*). As a control, a mutated 3′UTR of *IRS-1* gene (IRS1 3U*mut*) lacking the miR-126 binding site was used. (**C**) IRS1-3U*wt* (closed column) or IRS1-3U*mut* (open column) construct was cotransfected with the indicated concentration of empty plasmid (Control) or miR-126 expression plasmid (miR-126) into SK-Hep1 hepatocytes. Reporter gene assay were performed using a Dual-luciferase assays kit as described in Methods. The relative luciferase activities were plotted against that of control, which is set as 100%. Values are expressed as means ± SEM from four independent experiments. *, P<0.05; **, P<0.01 vs IRS1-3U*mut.*

Research findings have suggested that these up-regulated miRNAs may be translational repressors of IRS-1 expression, and that they may be involved in the development of insulin resistance. Although we selected miRNAs that are thought to target *IRS-1* 3′UTR, most of them have not yet been validated for whether they actually repress IRS-1 expression. Recently, Zhang *et al*. [32] found that miR-126 inhibited cell cycle progression in cancer cells via the targeting of *IRS-1* 3′UTR. We therefore selected miR-126 for further investigation of its functional relevance in the development of insulin resistance. To determine whether miR-126 directly regulates the expression of IRS-1 in SK-Hep1 hepatocytes, we set up an assay in which 414 nt 3′UTR of the *IRS-1* gene, which contains miR-126 binding site (Figure 5*A*–*B*), was inserted downstream of a luciferase open reading frame (IRS1 3U*wt*). A mutated 3′UTR of the *IRS-1* gene (IRS1 3U*mut*), which lacks miR-126 binding site, was used as a control (Figure 5*B*). Plasmid DNA of each pGL3-promoter-based 3′UTR reporter (IRS1-3U*wt* or IRS1-3U*mut*) was cotransfected with the indicated concentration of empty plasmid (pSilence6.1) or miR126 expression plasmid into SK-Hep1 hepatocytes. We found that luciferase activity with IRS1-3U*wt* was significantly inhibited by miR-126 in a dose-dependent manner, whereas luciferase activity with IRS1-3U*mut* was not (Figure 5*C*). These data suggest that miR-126 directly targets *IRS-1* 3′UTR and suppresses IRS-1 expression at the posttranscriptional level.

**Figure 6:**
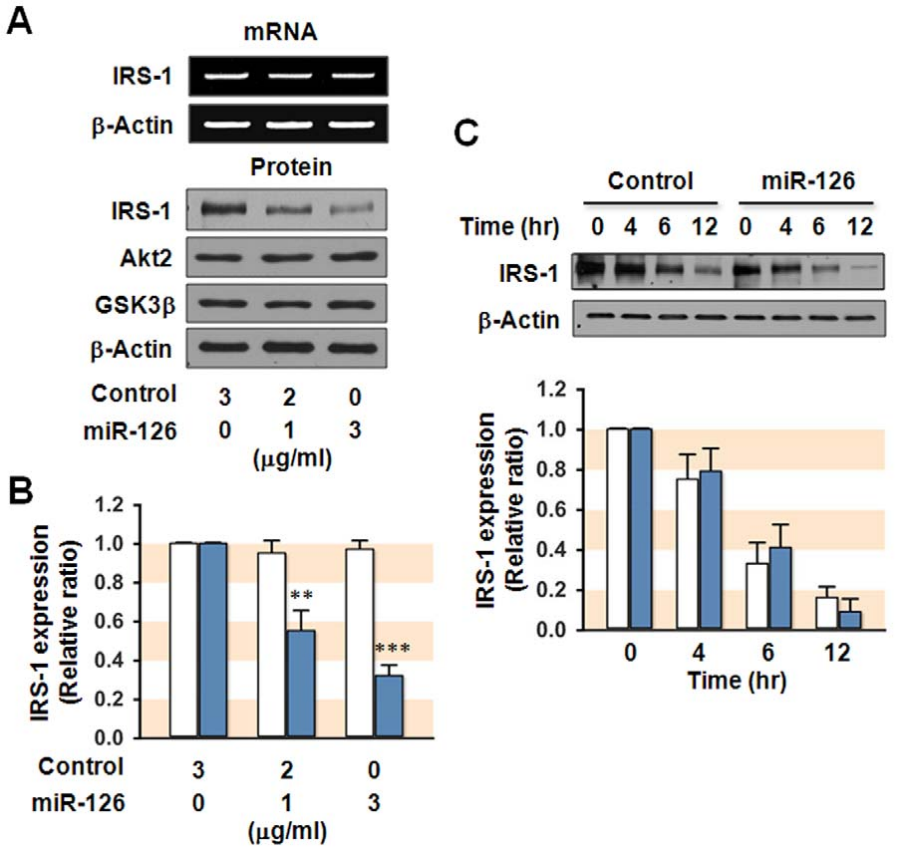
Effect of miR-126 on expression of IRS-1. SK-Hep1 hepatocytes were transfected with the indicated concentration of empty plasmid (Control) or miR-126 expression plasmid (miR-126). (**A**) mRNA level was analyzed 24 h of transfection, whereas immunoblot analysis was conducted after 72 h of transfection. These are representative results from five independent experiments. (**B**) Expressions of IRS-1 were analyzed by *q*RT-PCR (open column) and immunoblotting (closed column). The mRNA and protein of IRS-1 were expressed in relative ratio, where the intensity of empty plasmid alone was set to one. (**C**) SK-Hep1 hepatocytes were transfected with 3 µg/ml of empty plasmid (Control, open column) or miR-126 expression plasmid (miR-126, closed column). After 60 h of transfection, cells were treated with cycloheximide (50 µg/ml for 12 h) and total cell lysates (15 µg protein) were subjected to 8% SDS-PAGE. The protein levels of IRS-1 were analyzed by densitometry and expressed in relative ratio, where the intensity of 0 h was set to one. Values are expressed as means ± SEM from at least five independent experiments. **, P<0.01; ***, P<0.001.

To confirm that *IRS-1* is a target gene of miR-126 in hepatocytes, we transfected SK-Hep1 cells with empty plasmid or miR-126 expression plasmid, and investigated whether induction of miR-126 directly represses IRS-1 expression. As shown in Figure 6, the overexpression of miR-126 resulted in a substantial reduction in the level of IRS-1 protein with no apparent change in the level of IRS-1 mRNA, thereby indicating the posttranscriptional repression of IRS-1 expression by miR-126. However, the protein levels of Akt2 and GSK3β remained unaffected (Figure 6*A–B*). We also examined whether miR-126 could induce posttranslational degradation of IRS-1 in SK-Hep1 cells (Figure 6*C*). Cycloheximide, an inhibitor of peptidyl transferase in eukaryotic ribosome, is used for the analysis of IRS-1 degradation rate by blocking *de novo* synthesis of proteins. Although overexpression of miR-126 significantly reduced expression of IRS-1, the degradation rate of IRS-1 was not affected by miR-126. Therefore, it is suggested that the forced expression of miR-126 in hepatocytes does not affect the expression of IRS-1 posttranslationally.

**Figure 7:**
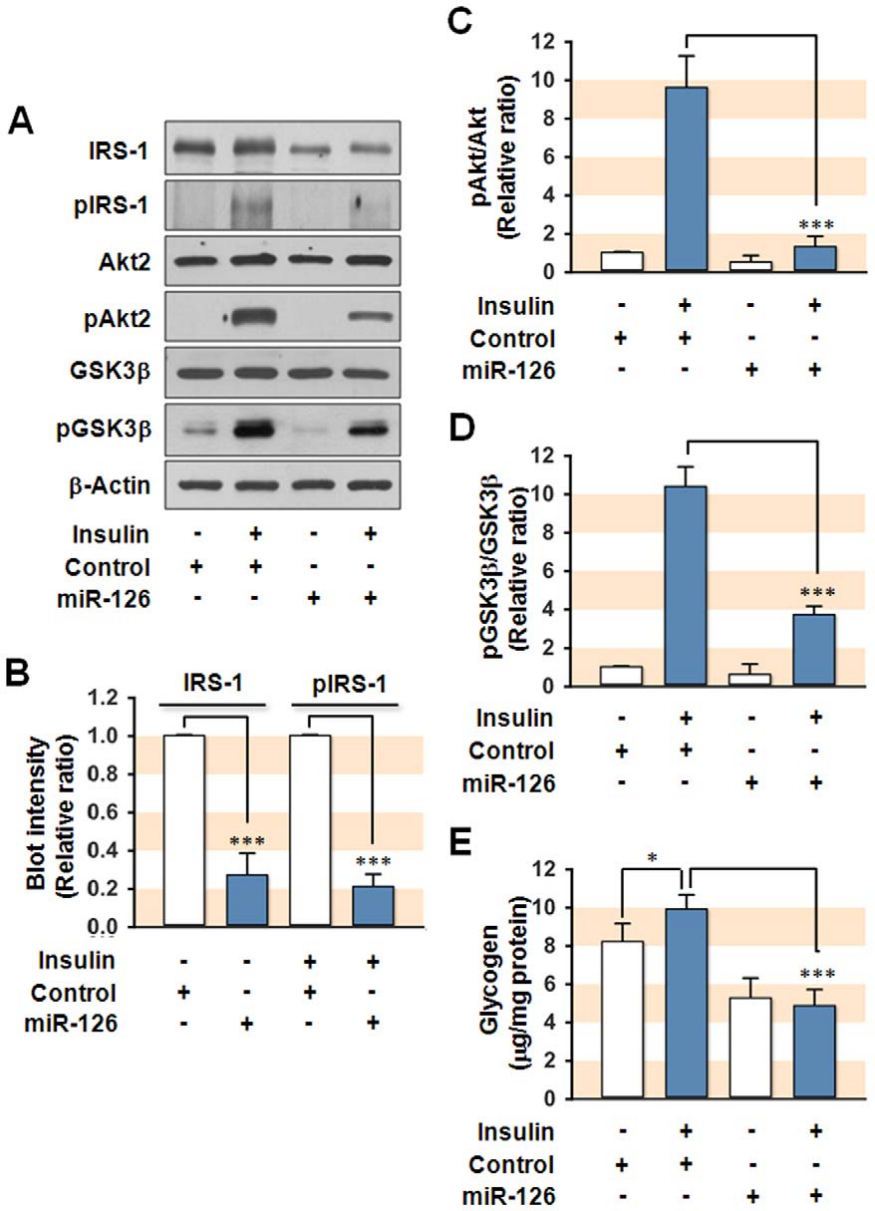
Effect of miR-126 on insulin signaling. SK-Hep1 hepatocytes were transfected with 3 µg/ml of empty plasmid (Control) or miR-126 expression plasmid (miR-126). After 72 h of transfection, cells were preincubated in the absence or presence of insulin (100 nM for 20 min) and total cell lysates (15 µg protein) were subjected to 8% SDS-PAGE. (**A**) The expression (IRS-1, Akt2, and GSK3β) and phosphorylation (pIRS-1, pAkt2, and pGSK3β) levels of insulin signaling intermediates were analyzed by immunoblot. (**B**) The immunoblot intensities for IRS-1/β-Actin (IRS-1) and insulin-stimulated pIRS-1/β-Actin (pIRS-1) were quantified by densitometry and expressed in relative ratio, where the intensity of normal control was set to one. (**C–D**) The immunoblot intensities for pAkt2/Akt2 and pGSK3β/GSK3β were quantified by densitometry and expressed in relative ratio. The intensity of normal control was set to one. (**E**) Cellular glycogen contents were analyzed by spectrophotometry (570 nm) with OxiRed probe. Values are expressed as means ± SEM from at least five independent experiments. *, P<0.05; ***, P<0.001.

Next, we analyzed expression and insulin-stimulated phosphorylation of insulin signaling intermediates in the hepatocytes transfected with miR-126 expression plasmid (Figure 7). The expression of IRS-1 was significantly reduced by 75% after miR-126 overexpression, whereas expression of Akt2 and GSK3β was not affected. As expected, the overexpression of miR-126 significantly reduced insulin-stimulated phosphorylation of IRS-1 and its downstream kinases, Akt2 and GSK3β in hepatocytes, and this effect was mainly due to a reduction in the expression of IRS-1 (Figure 7*B*–*D*). In addition, we determined how the overexpression of miR-126 affects cellular glycogen contents in the presence or absence of insulin (Figure 7*E*). In control cells, insulin significantly increased glycogen contents in hepatocytes. However, glycogen content in miR-126-overexpressed hepatocytes was significantly reduced as compared to control. Moreover, induction of miR-126 abolished insulin-stimulated glycogen synthesis (Figure 7*E*). Taken together, these data clearly indicate that the induction of miR-126 causes insulin resistance in hepatocytes through a reduction in the expression of IRS-1.

## Discussion

Mitochondrial dysfunction is associated with the development of insulin resistance and diabetes [3], [4], [16]. In the present study, we demonstrated that mitochondrial dysfunction resulting from genetic or metabolic inhibition induced the development of insulin resistance in hepatocytes via a reduction of IRS-1 expression. This result is in accordance with previous findings in myocytes that a reduced expression of IRS-1 is involved in the development of insulin resistance [18], [19]. Recent work in myocytes has revealed that mitochondrial retrograde signals inhibit the expression of IRS-1 at the transcriptional level by activating c-Jun N-terminal kinase (JNK) and p38 mitogen-activated protein kinase (MAPK), which in turn up-regulates an IRS-1 transcriptional suppressor, ATF3 [19]. Although the regulation of IRS-1 expression occurs at the transcriptional level under conditions of mitochondrial dysfunction, posttranscriptional regulation also occurs. For example, mitochondrial dysfunction activates certain stress kinases, such as IκB kinaseβ and JNK. These are well-known serine kinases that phosphorylate IRS-1 at serine residues, leading to decreased metabolic signaling by increasing IRS-1 degradation and serine phosphorylation [16]. Although coordinated alteration in expression of skeletal muscle miRNAs relates to insulin resistance in diabetes [33], the involvement of miRNAs in posttranscriptional repression of IRS-1 are largely unknown, especially in mitochondrial dysfunction,

The fact that no previous studies have investigated the role of miRNAs in insulin signaling accounts for the lack of research into their potential as therapeutic targets for metabolic disease. We hypothesized that certain miRNAs may participate in the development of insulin resistance induced by mitochondrial dysfunction. We observed a significant increase in the expression of several presumed *IRS-1*–3′UTR-targeting miRNAs in hepatocytes with dysfunctional mitochondria. In particular, we found that ectopic expression of miR-126, an induced *IRS-1*-targeting miRNAs, represses translation of IRS-1 without altering the mRNA level of IRS-1, which subsequently leads to insulin resistance in hepatocytes. Expression of miR-126 is decreased in various cancer cells, and it has previously been regarded as a cell growth suppressor that acts on IRS-1 [32], HOXA9 [34] and p85β [35]. In the present study, we have unveiled a new role of miR-126 as a metabolic regulator. Further, several other miRNAs predicted to target the *IRS-1* 3′UTR were induced by mitochondrial dysfunction in a similar fashion to miR-126, it is still open questions whether these miRNAs directly target the 3′UTR and repress IRS-1 expression. In addition, the expression of miRNA target genes can be modulated by altering the identity or the concentration of the miRNAs within cells [36]. Therefore, the combinational binding of different miRNAs on non-overlapping binding sites of *IRS-1* 3′UTR may synergistically potentiate the miRNA-induced repression of IRS-1. The discovery that miR-126 regulates insulin signaling provides novel insights into the molecular basis underlying mitochondrial dysfunction-derived pathogenesis of insulin resistance, and implicates miRNAs in metabolic diseases.

Here, we propose a possible mechanism for how miRNA participates in the development of insulin resistance under conditions of mitochondrial dysfunction. Mitochondrial dysfunction results in the generation of various retrograde signals to the nucleus, as described initially in myocytes [37]. These retrograde signals activate several kinases and transcriptional factors, resulting in the suppression of IRS-1 transcription and induction of IRS-1 degradation [19]. However, the results of present study suggest that some of the transcriptional factors that are affected by this retrograde signaling may activate the production and processing of miRNAs targeting the *IRS-1* 3′UTR, thereby leading to IRS-1 posttranscriptional repression and subsequent insulin resistance. Further analysis of which metabolic signals and transcriptional factors are involved and how they work in miRNA-mediated insulin resistance will be the next step of research.

In conclusion, we have demonstrated that mitochondrial dysfunction induced by mtDNA depletion or metabolic inhibition causes the development of insulin resistance in hepatocytes through a reduction in the expression of IRS-1. We found that mitochondrial dysfunction significantly up-regulated the expression of several miRNAs that are thought to target IRS-1. We confirmed that miR-126 is a crucial inhibitory factor in insulin signal transduction under conditions of mitochondrial dysfunction. Consequently, these data provide a novel mechanism for the development of insulin resistance, and serve as a foundation for further studies designed to explore the functional relevance of miRNAs in diabetes and metabolic syndrome.

## Materials and Methods

### Cell culture and induction of mitochondrial dysfunction

SK-Hep1 hepatocytes (ATCC HTB-52) were cultured in Dulbecco's Modified Eagle Medium (DMEM) with 10% fetal bovine serum (FBS). For mtDNA depletion, cells were incubated with EtBr (0.2 µg/ml) and uridine (50 µg/ml) for 2 weeks in DMEM with 10% FBS. Under these experimental conditions, mtDNA was depleted to <10% of normal. For mitochondrial metabolic inhibition, cells were treated with Rotenone (0.1 µM) or Antimycin A (20 µM) for 18 h. Hepatocytes were deprived of serum for 5 h prior to all experimental analysis.

### Genomic DNA extraction and polymerase chain reaction (PCR)

Total cellular DNA was extracted according to the manufacturer's instructions by using DNeasy Tissue kit (Qiagen). PCR and *q*RT-PCR was performed in LightCycler®480 (Roche-Applied Science) using SYBR-Green PCR Master Mix according to manufacturer's instructions (Qiagen) with the specific primers listed in Table S1.

### Staining of functional mitochondria

SK-Hep1 hepatocytes were incubated with the active mitochondrial-specific fluorescent dye, MitoTracker Orange CM-H_2_TMRos (Molecular Probes, Eugene, OR), for 15 min at 37°C and washed three times with cold PBS. The cells were visualized and photographed in a fluorescence microscope (Leica, Germany). The fluorescent intensity reflects the integrity of mitochondrial function [38].

### Measurement of cellular ATP levels

The cellular ATP was measured by using a somatic cell ATP assay kit (Sigma, Louis, MO) as described in the manufacturer's procedure.

### RNA preparation and quantitative real-time RT-PCR (qRT-PCR)

Total cellular RNA was extracted using miRNeasy Mini kit (Qiagen, Valencia, CA). For *q*RT-PCR, total RNAs were reverse transcribed into cDNAs with miScript Reverse Transcription kit (Qiagen) and amplified with specific primers (Bionics, Seoul, Korea) listed in Table S1. *q*RT-PCR was carried out in LightCycler®480 (Roche-Applied Science, Mannheim, Germany) using SYBR-Green PCR Master Mix according to manufacturer's instructions (Qiagen).

### Overexpression of miR-126 and reporter gene assay

Plasmids (pSilencer4.1CMV-puro, Ambion) expressing miR-126 pre-miRNA (flanking upstream and downstream 30–50 nt) for overexpression of miR-126 was produced as described previously [32] and transfected to SK-Hep1 hepatocytes with Lipofectamine 2000. For luciferase assay, DNA fragments (414 nt) of *IRS-1* 3′UTR containing predicted miR-126 binding site (IRS1-3U*wt*) were cloned into the pGL3-promoter plasmid (Promega) and the mir-126 binding sites were replaced with an 4 nt fragment to produce mutated 3′UTR pGL3 report plasmids (IRS1-3U*mut*) as described [32]. Luciferase assays were performed with the Dual luciferase reporter system (Promega) in accordance with manufacturer's instructions. Briefly, SK-Hep1 cells were plated the day before transfection onto 12-well plates and grown to an approximate 70% confluence. The cells were cotransfected with IRS1-3U*wt* or 3U*mut* and miR-126 expression plasmid together with pRL-SV40 for constitutive expression of *Renilla* luciferase as an internal control. At 48 h posttransfection, the cells were lysed and subjected to the measurement of the firefly and *Renilla* luciferase activities on a Lumat LB 9507 luminometer (EGG, Stuttgart, Germany). The ratio of firefly luciferase activity to *Renilla* luciferase activity was presented in arbitrary units as the relative luciferase activities.

### Glycogen Assay

Glycogen contents in the cultured cells were analyzed with Glycogen Assay Kit (BioVision, Mountain View, CA) by spectrophotometry (570 nm) with OxiRed probe according to manufacturer's suggestion.

### Gel Electrophoresis and Immunoblotting

Cell lysates solubilized in Laemmli solution were subjected to SDS-PAGE on 8 or 10% resolving gels and immunoblotting as described [39]. Antibody against IRS-1 was purchased from Upstate (Lake Placid, NY) and antibody against phosphor IRS-1 was from Invitrogen (Carlsbad, CA). All other antibodies were obtained from Cell Signaling (Beverly, MA). Proteins were visualized using enhanced chemiluminescent substrate kit (NEN Life Science Products). Immunoblot intensities were quantitated by densitometry using an analytical scanning system (Molecular Dynamics Inc., Sunnyvale, CA).

### Database and Statistical analysis

We computationally screened miRNAs targeting *IRS-1* 3′UTR with TargetScan (http://www.targetscan.org/index.html) and miRBase (http://microrna.sanger.ac.uk). Results were expressed as the mean ± SEM. Where applicable, the significance of difference was analyzed using Student's *t* test for unpaired data.

## Supporting Information

Figure S1 **Mitochondrial dysfunction induced by EtBr. **(**A**) Genomic DNA and mRNA were isolated from control or EtBr-treated SK-Hep1 hepatocytes, and mtDNA-encoded genes such as cytochrome c oxidase subunit I (COX-I) and subunit II (COX-II), and nuclear DNA-encoded gene such as cytochrome c oxidase subunit IV (COX-IV) were amplified by RT-PCR and *q*RT-PCR. (**B**) The relative values were expressed in arbitrary units where the intensity of control was set to one. (**C**) Control and EtBr-treated SK-Hep1 hepatocytes were stained with the active mitochondrial-specific fluorescent dye, MitoTracker Orange CM-H_2_TMRos, as described in Methods. Magnification is ∼ X400. (**D**) Total cellular ATP levels were measured by the luciferin/luciferase assay. ATP content was expressed in arbitrary units where the ATP content from control cells was set to one. All results represent mean ± SEM from three independent experiments. **, P<0.01; ***, P<0.001. **Results**: As shown in Figure S1 (A and B), cytochrome oxidase subunits I (COX-I) and II (COX-II), both encoded only in mtDNA, were hardly amplified from the genomic DNA and cDNA of the cells treated with EtBr for 2 weeks. In contrast, nuclear DNA-encoded genes such as COX-IV and β-actin were detected at similar levels in both control and EtBr-treated hepatocytes, indicating that prolonged treatment with EtBr depleted the cellular contents of mtDNA and its transcripts without altering the nuclear DNA replication. Next, we measured mitochondrial function with the active mitochondrial-specific fluorescent dye, MitoTracker Orange CM-H_2_TMRos [40], [41], [42], [43]. Since this Mitotracker localizes in mitochondria in a ΔΨ_m_-dependent manner, the fluorescent intensity from living cells thoroughly reflects the integrity of mitochondrial function. The depletion of mtDNA induced by EtBr treatment drastically reduced functional mitochondria in the cell (C). In addition, the depletion of mtDNA significantly reduced total cellular ATP level as compared to control cells (D). These data clearly indicates that cellular treatment with EtBr depleted mtDNA without altering nuclear DNA and provoked mitochondrial dysfunction in SK-Hep1 hepatocytes.
(TIF)

Figure S2 **Inhibition of mitochondrial function by Rotenone and Antimycin A.** For mitochondrial metabolic inhibition, cells were treated with Rotenone (0.1 µM) or Antimycin A (10 µM) for 18 h. Hepatocytes were deprived of serum for 5 h prior to all experimental manipulations. Cells were stained with the active mitochondrial-specific fluorescent dye, MitoTracker Orange CM-H_2_TMRos, as described in Methods. Magnification is ∼ X400.
(TIF)

Figure S3 **Expression of IRS-1 under ER stress induced by tunicamycin.** SK-Hep1 hepatocytes were treated with vehicle or tunicamycin for 18 hr, and mRNA and protein of IRS-1 were measured by RT-PCR and immunoblotting, respectively. Phosphorylation of eIF2α (peIF2α) was used for ER stress marker.
(TIF)

Table S1 **Primers and PCR conditions used in this study.**
(TIF)
