# Correction: The Induction of MicroRNA Targeting IRS-1 Is Involved in the Development of Insulin Resistance under Conditions of Mitochondrial Dysfunction in Hepatocytes

**DOI:** 10.1371/annotation/2faafaa7-e359-4711-af5b-3597c705388d

**Published:** 2011-05-11

**Authors:** Hyun Su Ryu, Seung-Yoon Park, Duan Ma, Jin Zhang, Wan Lee

The grant number from National Research Foundation of Korea is incorrect. The correct grant number is: "2009-0067910" 

