# Supplementary figures and images for: The Induction of MicroRNA Targeting IRS-1 Is Involved in the Development of Insulin Resistance under Conditions of Mitochondrial Dysfunction in Hepatocytes
Source: PLoS One. 2011 Mar 25;6(3):e17343. doi: 10.1371/journal.pone.0017343 (PMC3064581; doi:10.1371/journal.pone.0017343)

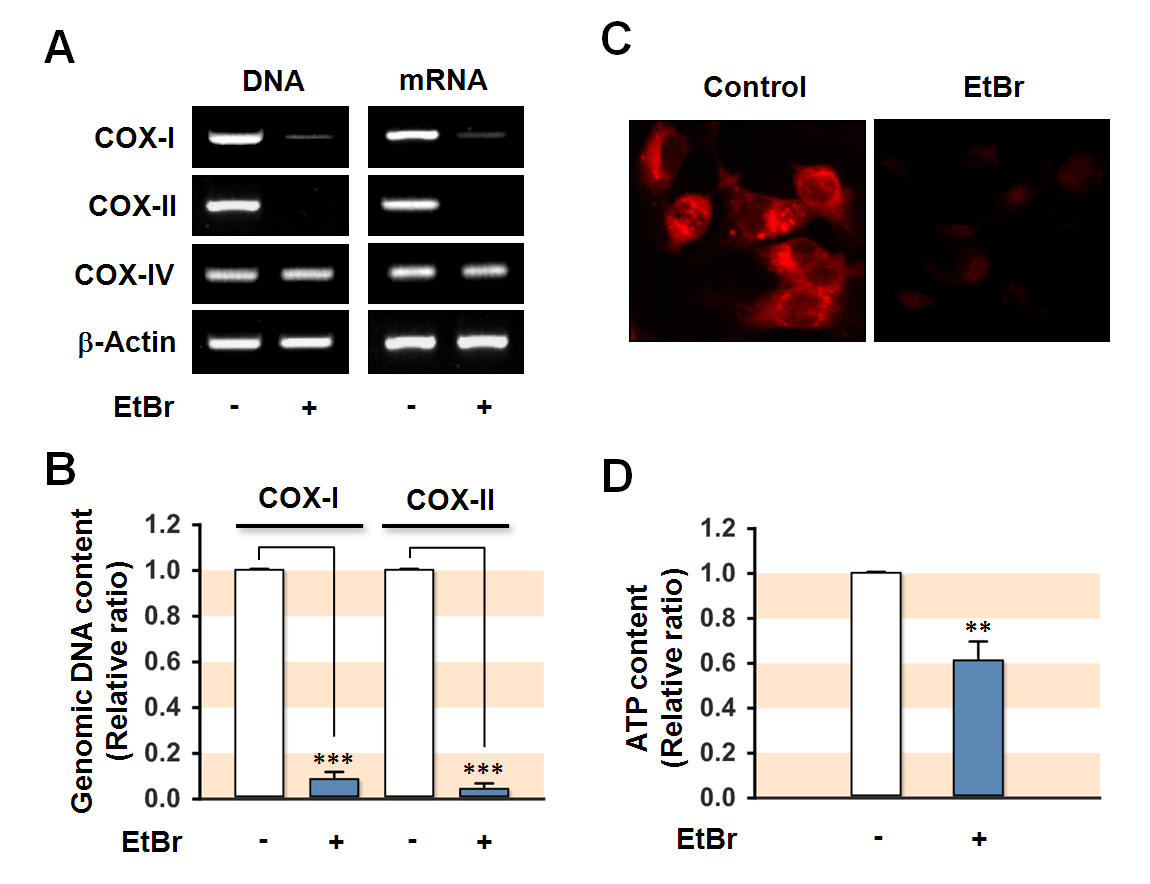

Supplement: Figure S1 — Mitochondrial dysfunction induced by EtBr. (A) Genomic DNA and mRNA were isolated from control or EtBr-treated SK-Hep1 hepatocytes, and mtDNA-encoded genes such as cytochrome c oxidase subunit I (COX-I) and subunit II (COX-II), and nuclear DNA-encoded gene such as cytochrome c oxidase subunit IV (COX-IV) were amplified by RT-PCR and qRT-PCR. (B) The relative values were expressed in arbitrary units where the intensity of control was set to one. (C) Control and EtBr-treated SK-Hep1 hepatocytes were stained with the active mitochondrial-specific fluorescent dye, MitoTracker Orange CM-H2TMRos, as described in Methods. Magnification is ∼ X400. (D) Total cellular ATP levels were measured by the luciferin/luciferase assay. ATP content was expressed in arbitrary units where the ATP content from control cells was set to one. All results represent mean ± SEM from three independent experiments. **, P<0.01; ***, P<0.001. Results: As shown in Figure S1 (A and B), cytochrome oxidase subunits I (COX-I) and II (COX-II), both encoded only in mtDNA, were hardly amplified from the genomic DNA and cDNA of the cells treated with EtBr for 2 weeks. In contrast, nuclear DNA-encoded genes such as COX-IV and β-actin were detected at similar levels in both control and EtBr-treated hepatocytes, indicating that prolonged treatment with EtBr depleted the cellular contents of mtDNA and its transcripts without altering the nuclear DNA replication. Next, we measured mitochondrial function with the active mitochondrial-specific fluorescent dye, MitoTracker Orange CM-H2TMRos [40], [41], [42], [43]. Since this Mitotracker localizes in mitochondria in a ΔΨm-dependent manner, the fluorescent intensity from living cells thoroughly reflects the integrity of mitochondrial function. The depletion of mtDNA induced by EtBr treatment drastically reduced functional mitochondria in the cell (C). In addition, the depletion of mtDNA significantly reduced total cellular ATP level as compared to control cells (D). [file pone.0017343.s001.tif]

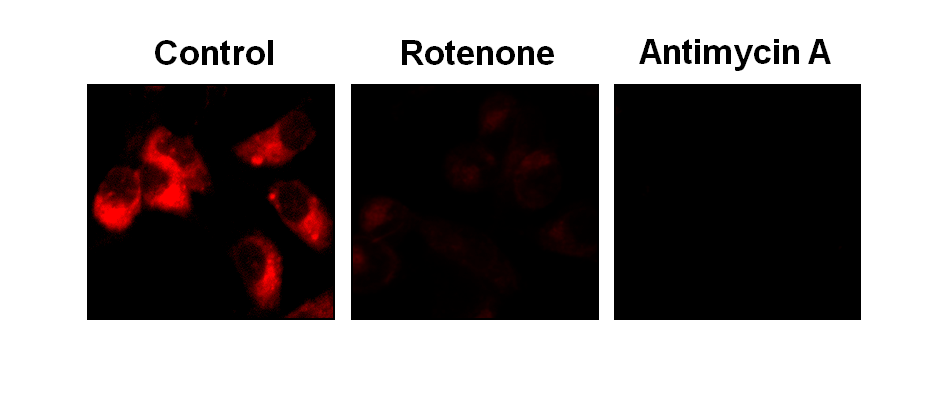

Supplement: Figure S2 — Inhibition of mitochondrial function by Rotenone and Antimycin A. For mitochondrial metabolic inhibition, cells were treated with Rotenone (0.1 µM) or Antimycin A (10 µM) for 18 h. Hepatocytes were deprived of serum for 5 h prior to all experimental manipulations. Cells were stained with the active mitochondrial-specific fluorescent dye, MitoTracker Orange CM-H2TMRos, as described in Methods. Magnification is ∼ X400. (TIF) [file pone.0017343.s002.tif]

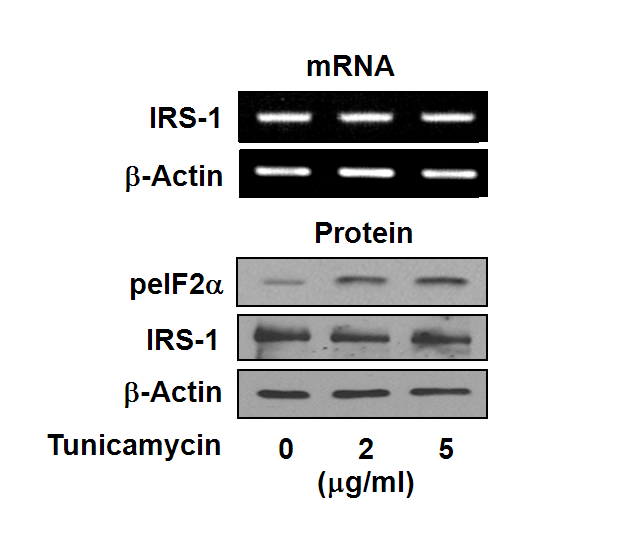

Supplement: Figure S3 — Expression of IRS-1 under ER stress induced by tunicamycin. SK-Hep1 hepatocytes were treated with vehicle or tunicamycin for 18 hr, and mRNA and protein of IRS-1 were measured by RT-PCR and immunoblotting, respectively. Phosphorylation of eIF2α (peIF2α) was used for ER stress marker. (TIF) [file pone.0017343.s003.tif]

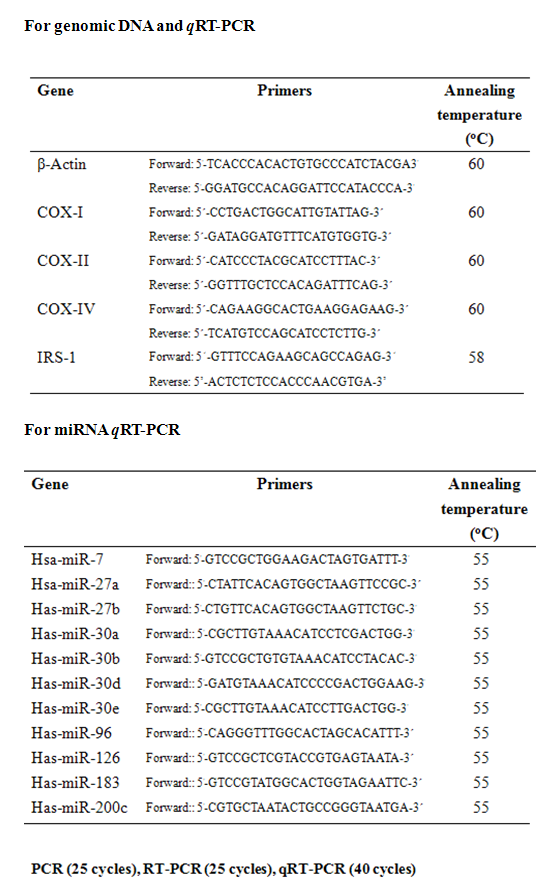

Supplement: Table S1 — Primers and PCR conditions used in this study. (TIF) [file pone.0017343.s004.tif]
